# Cold-Induced Elevation of 3-Hydroxypropionate Exacerbates Colitis by Remodeling Gut Microbiota and Impairing Mitochondrial Respiration in Intestinal Epithelial Cells

**DOI:** 10.3390/metabo16080592

**Published:** 2026-08-19

**Authors:** Yankun Jia, Baodong Gao, Kefei Wu, Mengjie Gao, Qi Lin, Tu Qian, Junjie Ma, Hongyu Zhang, Ping Zhu, Zhinan Chen, Yue Zhai

**Affiliations:** 1 Department of Cell Biology and National Translational Science Center for Molecular Medicine, Fourth Military Medical University, Xi’an 710032, China; 18234706092@163.com (Y.J.); dongbbyy@163.com (B.G.); wkfworkemail@163.com (K.W.);; 2 National Infrastructure for Translational Medicine (Xi’an), Xi’an 710032, China; 3 State Key Laboratory of New Targets Discovery and Drug Development for Major Diseases, Xi’an 710032, China; 4 Department of Clinical Immunology, Xijing Hospital, Fourth Military Medical University, Xi’an 710032, China

**Keywords:** gut microbiota, 3-hydroxypropionate, cold exposure, colitis, microbial metabolism, short-chain fatty acids, mitochondrial respiration, tight junctions

## Abstract

Background/Objectives: Inflammatory bowel disease (IBD) is a chronic gastrointestinal disorder influenced by environmental factors including cold stress. While cold exposure exacerbates intestinal inflammation, the specific microbial metabolites linking environmental stress to colitis remain unclear. 3-Hydroxypropionate (3-HPA) is a gut microbial metabolite elevated following cold exposure, but its pathogenic role in intestinal inflammation has not been investigated. This study aimed to determine whether 3-HPA contributes to colitis progression and to characterize its effects on gut microbiota and intestinal epithelial function. Methods: We employed a dextran sulfate sodium (DSS)-induced colitis mouse model to assess the impact of cold exposure and exogenous 3-HPA administration. Paired shotgun metagenomic and metabolomic analyses were performed to evaluate gut microbial composition and metabolic outputs. Mechanistic studies using NCM460 intestinal epithelial cells were conducted to examine mitochondrial respiration and tight junction integrity under nutrient-limited conditions. Results: Cold exposure increased fecal 3-HPA levels and aggravated DSS-induced colitis, characterized by enhanced weight loss, histological damage, and immune cell infiltration. Direct 3-HPA supplementation alone was sufficient to worsen colitis severity. Multi-omics profiling revealed that 3-HPA reshaped gut microbiota composition, depleted short-chain fatty acids (SCFAs), and disrupted microbial tryptophan and bile acid metabolism. In vitro, 3-HPA impaired mitochondrial oxidative phosphorylation, reduced ATP production, and compromised tight junction organization in intestinal epithelial cells. Conclusions: These findings identify 3-HPA as a gut microbial metabolite elevated by cold exposure that contributes to colitis progression by disrupting beneficial microbial metabolism while also impairing epithelial mitochondrial function and barrier integrity. Modulating 3-HPA production or its downstream epithelial effects may represent a potential therapeutic approach for IBD exacerbated by environmental stress.

## 1. Introduction

Inflammatory bowel disease (IBD), encompassing ulcerative colitis (UC) and Crohn’s disease (CD), is a chronic relapsing inflammatory disorder of the gastrointestinal tract with a rapidly increasing global incidence [1]. Although its pathogenesis involves complex interactions among genetic susceptibility, immune dysregulation, intestinal barrier dysfunction, and environmental influences, growing evidence suggests that environmental stressors can substantially modify disease progression [2,3]. Among these factors, cold exposure has emerged as a potential aggravating factor in intestinal inflammation. Experimental studies have shown that cold stress exacerbates colitis and alters gut microbial composition [4,5]. However, the molecular mediators that translate cold-induced microbial alterations into intestinal pathology remain poorly understood.

The gut microbiota regulates intestinal homeostasis largely through its metabolic activities. Microbiota-derived metabolites act as critical mediators of host–microbe interactions, influencing epithelial function, immune responses, and mucosal integrity [6]. Among these, short-chain fatty acids (SCFAs) provide essential energy substrates for colonocytes and exert anti-inflammatory effects, whereas tryptophan-derived indole metabolites support epithelial barrier function and mucosal immunity through activation of aryl hydrocarbon receptor (AhR) signaling [7,8]. Disruption of these metabolic networks has been consistently linked to IBD pathogenesis [9,10]. Despite increasing recognition of the microbiota–metabolite–host axis, the specific metabolites induced by environmental stress that actively drive intestinal inflammation have yet to be defined. 3-Hydroxypropionate (3-HPA) is a short-chain hydroxy acid generated through microbial metabolic pathways and has recently emerged as a biologically active mediator of host–microbe interactions [11,12]. Notably, our recent study demonstrated that 3-HPA produced by Klebsiella and Escherichia coli can modify host proteins through cysteine carboxyethylation, which generated neoantigens that drive HLA-restricted autoimmunity in ankylosing spondylitis [12]. These findings establish 3-HPA as a microbiota-derived metabolite capable of directly influencing host pathogenic immune responses. However, whether 3-HPA contributes to intestinal inflammation, alters gut microbial ecology, or affects intestinal epithelial function has not been investigated. In our preliminary metabolomic analysis, we identified 3-HPA as a significantly elevated metabolite following acute cold exposure. These findings suggest that 3-HPA might act as a microbial metabolite that responds to cold, linking environmental stress to gut inflammation, which needs further investigation. Given the central role of microbial metabolites in maintaining epithelial homeostasis and energy metabolism, we further speculated that elevated 3-HPA might contribute to colitis progression through coordinated effects on the gut microbiota and intestinal epithelial cells.

Here, we investigated the role of 3-HPA in dextran sulfate sodium (DSS)-induced colitis using in vivo models, multi-omics analyses, and mechanistic cellular experiments. Together, our findings identify 3-HPA as a cold-elevated gut microbial metabolite whose exogenous supplementation exacerbates colitis, highlighting its potential role as a metabolic link between environmental stress and intestinal inflammation.

## 2. Materials and Methods

### 2.1. Animal Experiments

Specific pathogen-free grade C57BL/6 N mice (6–8 weeks old, male) were purchased fromTengxin Biotechnology Co., Ltd. (Chongqing, China), and were housed under a 12 h light/12 h dark cycle at a constant temperature (22 °C) with free access to food and water. After 7 days of adaptive feeding, mice were randomly assigned to experimental groups. Regarding the influence of sex, only male mice were used in this study. As no cross-sex comparative analyses were performed, the potential impact of sex on the reported outcomes remains undetermined, which represents a limitation regarding the generalizability of the conclusions. We anesthetized the mice with 3% isoflurane via the respiratory pathway for 2 min and performed cervical dislocation to collect the colon, spleen, liver, and fecal contents.

#### 2.1.1. Cold Exposure Combined with DSS-Induced Colitis

Mice were randomly divided into three groups: Blank group (*n*= 10, normal drinking water, room temperature), DSS group (*n* = 10, 2% DSS in drinking water, room temperature), and DSS + CE group (*n* = 10, 2% DSS in drinking water, cold exposure). Dextran sulfate sodium (DSS; molecular weight 35,000–50,000; cat. no. 216011090; MP Biomedicals, Solon, OH, USA) was dissolved in drinking water at a concentration of 2% (*w*/*v*) and provided to the mice ad libitum for 8 consecutive days, with fresh DSS solution replaced every 2 days, according to established murine colitis induction protocols. Cold exposure was performed by placing mice in a cold chamber at 4 °C for 5 h per day, during which food and water were withheld. The experimental protocol consisted of 8 days of DSS administration with concomitant cold exposure, followed by a 5-day resting phase with normal drinking water.

Animal welfare and body weights were rigorously monitored on a daily basis. In accordance with established humane endpoints approved by our institutional animal welfare protocol, mice were immediately euthanized if they exhibited a loss of ≥20% of their initial body weight, gross rectal bleeding, or critical signs of distress (e.g., severe lethargy, hunched posture, or loss of righting reflex during the cold challenge).

#### 2.1.2. 3-HPA Supplementation During DSS-Induced Colitis

Mice were randomly divided into three groups: Blank group (*n* = 6, normal drinking water), DSS group (*n* = 6, 1.5% DSS in drinking water), and DSS + 3-HPA group (*n* = 6, 1.5% DSS plus 5 mM 3-HPA in drinking water). 3-Hydroxypropionate (3-HPA; cat. no. H823102; Macklin, Shanghai, China) was dissolved in drinking water at a final concentration of 5 mM, with the pH adjusted to approximately 7.0 using 1 M NaOH at a ratio of 2:5 (3-HPA: NaOH, *v*/*v*). Mice received 1.5% DSS dissolved in their drinking water ad libitum for 8 consecutive days (with fresh solution changed every 2 days) to induce mild-to-moderate colitis, followed by a 6-day resting phase accompanied by continuous 3-HPA administration [13,14]. Body weight was monitored daily.

A moderate concentration of 1.5% DSS was specifically chosen for the 3-HPA supplementation experiment (compared to the 2.0% DSS used in the cold-exposure study) to establish a baseline of mild-to-moderate colitis. This design prevented the masking of phenotypes by severe ulceration or early animal lethality and allowed a sensitive assessment of the additive toxic and disease-aggravating effects of continuous exogenous 3-HPA administration.

### 2.2. Histopathological Analysis

Colonic tissue was fixed in 4% paraformaldehyde, embedded in paraffin, and cut into 5-μm sections. H&E staining was performed by a commercial service (Yike Biotechnology, Xi’an, China). Histopathological scoring was independently performed by three blinded observers according to a validated murine colitis histological scoring system [15]. The scoring was based on four parameters, each graded on a scale of 0 to 3: (i) inflammatory cell infiltration (0: none; 1: mild infiltration in the mucosa; 2: moderate infiltration extending to the submucosa; 3: severe transmural infiltration); (ii) mucosal damage (0: intact epithelium; 1: mild epithelial disruption; 2: moderate epithelial erosion; 3: extensive epithelial ulceration); (iii) crypt architecture disruption (0: intact crypts; 1: mild crypt distortion; 2: moderate crypt loss; 3: complete crypt destruction); and (iv) edema and erosion (0: none; 1: mild submucosal edema; 2: moderate edema with focal erosion; 3: severe edema with extensive erosion). The sum of the four subscores yielded a total histological score ranging from 0 to 12.

### 2.3. Shotgun Metagenomic Sequencing and Functional Annotation

Shotgun metagenomic sequencing and bioinformatic analysis were performed by a commercial service (Heyuan Biotechnology, Shanghai, China). Briefly, total genomic DNA was extracted from fecal samples, and metagenomic libraries were constructed using the company’s standard commercial kits and protocols. Libraries were sequenced on the the Illumina NovaSeq platform (Illumina, San Diego, CA, USA) with paired-end 150 bp reads.

Raw sequencing reads were quality-filtered using Cutadapt (v1.9.1) to remove adapter sequences, low-quality bases, and reads with excessive N content. Host-derived reads were removed by mapping against the mouse reference genome (mm10) using BWA (v0.7.12). The remaining high-quality reads were assembled into contigs using MEGAHIT (v1.2.9) based on de Bruijn graph construction. Protein-coding genes were predicted from assembled contigs using Prodigal (v3.02). A non-redundant gene catalog was constructed using MMseqs2 (v11.e1a1c) with a sequence identity threshold of 95% and a coverage threshold of 95%. Gene abundances were calculated by mapping clean reads back to the gene catalog using SoapAligner (v2.21).

For functional annotation, predicted amino acid sequences were aligned against the Kyoto Encyclopedia of Genes and Genomes (KEGG) database using DIAMOND (v0.8.15.77) with an E-value cutoff of 1 × 10^−5^. The relative abundances of KEGG pathways at Level 3 were calculated by summing the abundances of genes annotated to each pathway. Taxonomic annotation was performed by aligning sequences against the NCBI non-redundant (NR) database using DIAMOND (v0.8.15.77), with taxonomic assignment based on the lowest common ancestor (LCA) algorithm. Alpha diversity indices (Chao1 and Shannon) were calculated using QIIME (v1.9.1).

### 2.4. Metabolomic Profiling

#### 2.4.1. Cold Exposure and Fecal Targeted Metabolomic Profiling

Mice were randomly divided into three experimental groups: the 37 °C group (maintained at room temperature), the 35–37 °C group, and the 32 °C group. For the cold challenge, mice in the latter two groups were placed in a cold chamber at 4 °C with food and water withheld. The cold exposure protocol was performed under strict monitoring. During the 4 °C challenge, the core body temperature of each mouse was monitored using a rectal probe. Mice were removed from the cold chamber immediately upon reaching their target core body temperatures (35–37 °C or <32 °C, respectively) for subsequent sampling. In accordance with our approved animal welfare protocol, any mice exhibiting signs of distress prior to reaching the target temperatures, or those with a core body temperature dropping below 30 °C, were immediately removed from the chamber and excluded from the experimental cohort.

All samples were immediately snap-frozen in liquid nitrogen and stored at −80 °C until metabolomic analysis. Absolute quantitative metabolomic profiling of fecal samples was performed by Metabo-Profile Biotechnology (Shanghai, China). Briefly, approximately 5 mg of lyophilized fecal material was weighed and transferred into a 1.5 mL tube. Samples were thawed on ice and homogenized with 25 μL of deionized water using zirconium beads. Ice-cold methanol containing internal standards (120 μL) was then added, followed by vortex homogenization. Samples were centrifuged at 18,000× *g* for 20 min at 4 °C. The supernatant (20 μL) was transferred to a 96-well plate for subsequent processing. Metabolites were derivatized using freshly prepared derivatization reagents at 30 °C for 60 min. After derivatization, samples were diluted with 330 μL of ice-cold 50% methanol and centrifuged at 4000× *g* for 30 min at 4 °C. Then, 135 μL of the supernatant was transferred to a new 96-well plate, and 10 μL of internal standard solution was added to each well.

Targeted metabolomic analysis was performed using an ACQUITY UPLC system coupled to a Xevo TQ-S tandem mass spectrometer (Waters Corp., Milford, MA, USA). Chromatographic separation was carried out on an ACQUITY UPLC BEH C18 column (2.1 mm × 100 mm, 1.7 μm) equipped with a VanGuard pre-column (2.1 mm × 5 mm, 1.7 μm). The mobile phase consisted of water containing 0.1% formic acid (A) and acetonitrile: isopropanol (70:30, *v*/*v*) (B). The gradient elution program followed the Q300 fecal metabolomics platform protocol. The mass spectrometer was operated in multiple reaction monitoring (MRM) mode for highly sensitive and selective detection of targeted metabolites. Raw data were acquired and processed using MassLynx software (v4.1, Waters) for peak integration, calibration, and absolute quantification. Subsequent statistical analysis and visualization were performed using the iMAP platform (v1.0, Metabo-Profile).

#### 2.4.2. 3-HPA Supplementation and Fecal Targeted Metabolomic Profiling

Healthy mice were randomly assigned to two experimental groups (*n* = 10 per group): a Control group receiving normal drinking water, and a 3-HPA group receiving 5 mM 3-HPA in their drinking water for a duration of 2 weeks.

Metabolomic profiling of the fecal samples was conducted by BioTree Biotechnology (Shanghai, China). Briefly, the samples were homogenized in a pre-chilled extraction solvent (methanol:acetonitrile:water) and centrifuged. The resulting supernatant was analyzed using a Vanquish ultra-high-performance liquid chromatography (UHPLC) system coupled to an Orbitrap Exploris 120 mass spectrometer (Thermo Fisher Scientific (Waltham, MA, USA)). Chromatographic separation was achieved on an ACQUITY UPLC BEH Amide column (2.1 mm × 100 mm, 1.7 μm). The mobile phase comprised 25 mmol/L ammonium acetate and 25 mmol/L ammonium hydroxide in water (pH 9.75) paired with acetonitrile. The mass spectrometer was operated in full-scan mode with data-dependent acquisition (DDA) to obtain MS/MS spectra. For data processing, raw files were converted into mzXML format using ProteoWizard (v3.0) and processed with XCMS software (v3.2) for peak detection, alignment, and integration. General metabolite annotation was performed by matching the acquired MS/MS spectra against an in-house standard database within a mass tolerance of 10 ppm. Furthermore, for the specific relative quantification of short-chain fatty acids (acetate, propionate, butyrate, and valerate) and tryptophan metabolites (tryptophan, indole-3-acetic acid, 3-indolepropionic acid, 3-indoleacrylic acid, and indole-3-lactic acid), extracted ion chromatograms were generated based on their exact masses, and the corresponding peak areas were precisely integrated using XCMS (v3.2).

### 2.5. Cell Culture and Treatments

The human normal colon epithelial cell line NCM460 was cultured in RPMI-1640 medium (Gibco, Waltham, MA, USA) supplemented with 10% fetal bovine serum (FBS, Gibco, Waltham, MA, USA) and 1% penicillin-streptomycin at 37 °C in a humidified atmosphere containing 5% CO_2_.

3-Hydroxypropionate (3-HPA; cat. no. H823102; Macklin, Shanghai, China), a liquid compound, was freshly prepared as a 1 M stock solution by adjusting the pH to approximately 7.0 using 1 M NaOH at a ratio of 2:5 (3-HPA: NaOH, *v*/*v*). The stock solution was further diluted in complete culture medium to a final concentration of 5 mM for treatment. For the control group, an equal volume of phosphate-buffered saline (PBS) was added to the complete culture medium, serving as the volume-matched vehicle control (designated as PBS). For in vitro cell treatments, 3-HPA was used at 5 mM. This concentration matches that administered to mice in the drinking water model and was chosen to approximate the locally elevated metabolite levels that intestinal epithelial cells may encounter at the mucosal–luminal interface during dysbiosis, which can exceed those in bulk fecal contents (0.1–1.0 mM) and peripheral serum (tens of μM). For experiments, cells were assigned to the following groups: PBS (vehicle control), 3HPA, Starvation (serum-free medium for 12 h), and Starvation+3HPA. For fatty acid oxidation (FAO) inhibition experiments in Seahorse analysis, etomoxir (Agilent Technologies, Santa Clara, CA, USA) was preloaded into Port A of the Seahorse sensor cartridge and injected at a final concentration of 40 μM at the start of the assay.

### 2.6. Cell Viability Assay

Cell viability was assessed using the Cell Counting Kit-8 (CCK-8; cat. no. E1CK-00208; Enogene, Nanjing, China) according to the manufacturer’s instructions. NCM460 cells were seeded in 96-well plates and treated as described above. At the end of the treatment period, 10 μL of CCK-8 solution was added to each well, and the plates were incubated at 37 °C for 30 min. The absorbance was measured at 450 nm using a Cytation 5 multimode imaging microplate reader (BioTek Instruments, Winooski, VT, USA).

### 2.7. RNA Extraction and Quantitative Real-Time PCR

Total RNA was extracted from NCM460 cells using the E.Z.N.A. Total RNA Kit (Omega Bio-Tek, R6934-01, Norcross, GA, USA) following the manufacturer’s protocol. RNA concentration and purity were determined using a NanoDrop 2000c spectrophotometer (Thermo Scientific, Wilmington, DE, USA). Complementary DNA (cDNA) was synthesized from 500 ng of total RNA using PrimeScript RT Master Mix (Perfect Real Time) (TAKARA, RR036A, Kusatsu, Shiga, Japan) according to the manufacturer’s instructions. Quantitative real-time PCR was performed on the QuantStudio 7 Real-Time PCR System (Applied Biosystems, Foster City, CA, USA) using TB Green Premix Ex Taq II (Tli RNase H Plus) (cat. no. RR820A; Takara Bio, Shiga, Japan). The primer sequences were as follows:

*GAPDH* forward: GCACCGTCAAGGCTGAGAAC

*GAPDH* reverse: TGGTGAAGACGCCAGTGGA

*HADHA* forward: CTGCCCAAAATGGTGGGTGT

*HADHA* reverse: GGAGGTTTTAGTCCTGGTCCC

*ACADVL* forward: ACAGATCAGGTGTTCCCATACC

*ACADVL* reverse: CTTGGCGGGATCGTTCACTT

*CPT1A* forward: TCCAGTTGGCTTATCGTGGTG

*CPT1A* reverse: TCCAGAGTCCGATTGATTTTTGC

*ZO-1* forward: GCATCATCAACCTCTGCTGTTAAT

*ZO-1* reverse: GCCTCATCATTTCCTCGGGATAT

Relative gene expression was calculated using the 2^−ΔΔCt^ method with GAPDH as the endogenous control.

### 2.8. Western Blot Analysis

Cells were lysed in RIPA Lysis Buffer (cat. no. P0013B; Beyotime, Shanghai, China). Equal amounts of protein (20 μg) were separated by SDS-PAGE using the CFAS any KD PAGE Protein Electrophoresis Gel Preparation Kit (Zhonghui Hecai, Xi’an, China, PE008) and transferred onto PVDF membranes (MilliporeSigma, Burlington, MA, USA). Membranes were blocked with 5% non-fat milk in TBST for 1 h at room temperature and then incubated overnight at 4 °C with primary antibodies: anti-occludin (Proteintech, Wuhan, China, 66378-1-Ig, 1:10,000), anti-claudin-1 (Proteintech, Wuhan, China, 13050-1-AP, 1:4000), anti-Actin (Proteintech, Wuhan, China, 66009-1-Ig, 1:10,000) and anti-GAPDH (Proteintech, Wuhan, China, 60004-1-Ig, 1:10,000). After washing with TBST, membranes were incubated with HRP-conjugated goat anti-mouse IgG (H+L) secondary antibody (Pierce Biotechnology, Rockford, IL, USA, 31430, 1:10,000) for 1 h at room temperature. Protein bands were visualized using the Ultra-sensitive ECL Chemiluminescence Kit (Xinsaimei, Suzhou, China, P10100) and imaged using a Bio-Rad ChemiDoc imaging system (Bio-Rad, Hercules, CA, USA). Band intensities were quantified using ImageJ software (v1.53, NIH).

### 2.9. Flow Cytometry Analysis

Spleens were mechanically dissociated into single-cell suspensions in RPMI-1640 medium. After red blood cell lysis using Ammonium-Chloride-Potassium (ACK) lysis buffer (TIANGEN, RT122-02, Beijing, China), cells were washed and resuspended in fluorescence-activated cell sorting (FACS) buffer (phosphate-buffered saline (PBS) containing 2% FBS). Single-cell suspensions were incubated with Fc block (anti-mouse CD16/CD32, BioLegend, 101319, San Diego, CA, USA) for 15 min at 4 °C, followed by staining with fluorochrome-conjugated antibodies against CD45 (PerCP/Cyanine5.5, BioLegend, 103131), CD11b (PE, BioLegend, 101207), F4/80 (APC/Cyanine7, BioLegend, 123118), and Ly-6G/Ly-6C (Gr-1, Brilliant Violet 421, BioLegend, 108445) for 30 min at 4 °C in the dark. After staining, cells were washed twice and resuspended in FACS buffer. Flow cytometric data were acquired on a CytoFLEX S multicolor analytical flow cytometer (Beckman Coulter, Brea, CA, USA) and analyzed using FlowJo software (v10, BD Biosciences, San Jose, CA, USA). Cells were gated sequentially by FSC-H/FSC-A (singlet discrimination), SSC-H/FSC-H (live cells), and CD45^+^ (total immune cells). Within the CD45^+^ gate, macrophages and dendritic cells were first excluded (F4/80^−^ CD11c^−^). From the remaining population, CD11b^+^ cells were selected. Since the Gr-1 antibody recognizes both Ly6G and Ly6C, the Gr-1 fluorescence signal was plotted against side scatter (SSC) to differentiate myeloid subsets. Neutrophils were identified as Gr-1hi SSChi, and monocytes as Gr-1int/low SSClow.

Peripheral blood was collected at the experimental endpoint into EDTA-coated tubes. After red blood cell lysis, the remaining leukocytes were stained with the same antibody panel and analyzed using the same gating strategy as described for splenocytes.

### 2.10. Serum Cytokine Quantification

Blood samples were allowed to clot and centrifuged at 3000× *g* for 15 min at 4 °C to collect serum. Serum concentrations of IL-6, IL-1β, and TNF-α were measured using precoated mouse ELISA kits (IL-6, cat. no. 1210602; IL-1β, cat. no. 1210122; TNF-α, cat. no. 1217202; all from Dakewe, Shenzhen, China) according to the manufacturer’s instructions.

### 2.11. Seahorse Analysis

NCM460 cells were seeded at 10,000 cells/well in Seahorse XF96 cell culture microplates (Agilent Technologies, Santa Clara, CA, USA) and allowed to adhere overnight. Cells were treated with or without 3-HPA as described above.

On the day of the assay, the culture medium was replaced with Seahorse XF DMEM Medium (Agilent Technologies, 103575-100, Santa Clara, CA, USA) supplemented with 10 mM glucose, 1 mM pyruvate, and 2 mM glutamine (for the mitochondrial stress test) or 2 mM glutamine (for the glycolysis stress test), and the pH was adjusted to 7.4. Cells were incubated in a non-CO_2_ incubator at 37 °C for 45–60 min before measurement.

### 2.12. Mitochondrial Stress Test

Oxygen consumption rate (OCR) was measured under basal conditions and after sequential injection of oligomycin (1.5 μM), FCCP (carbonyl cyanide-4-(trifluoromethoxy)phenylhydrazone) (1.0 μM), and rotenone/antimycin A (0.5 μM), according to the manufacturer’s recommended concentrations. Basal respiration was calculated as (basal OCR − non-mitochondrial OCR). Maximal respiration was calculated as (FCCP-stimulated OCR − non-mitochondrial OCR). Spare respiratory capacity was calculated as (maximal respiration − basal respiration). ATP production was calculated as (basal OCR − oligomycin-inhibited OCR). Non-mitochondrial oxygen consumption was measured as the OCR remaining after rotenone/antimycin A injection.

### 2.13. Glycolysis Stress Test

Extracellular acidification rate (ECAR) was measured under basal conditions and after sequential injection of glucose (10 mM), oligomycin (1.0 μM), and 2-deoxyglucose (50 mM), according to the manufacturer’s recommended concentrations. Glycolysis was calculated as (ECAR after glucose injection − non-glycolytic acidification). Glycolytic capacity was calculated as (ECAR after oligomycin injection − non-glycolytic acidification). Glycolytic reserve was calculated as (glycolytic capacity − glycolysis). Non-glycolytic acidification was measured as the ECAR remaining after 2-deoxyglucose injection.

### 2.14. FAO-Dependent Respiration Assay

For assessment of the contribution of fatty acid oxidation (FAO) to mitochondrial respiration, etomoxir (Agilent Technologies) was preloaded into Port A of the sensor cartridge at a final concentration of 40 μM and injected at the beginning of the assay, before the mitochondrial stress test protocol described above. OCR measurements and calculations were performed as described for the mitochondrial stress test.

All Seahorse data were normalized to protein content per well, determined by bicinchoninic acid (BCA) assay after completion of the Seahorse run.

### 2.15. Statistical Analysis

Data are expressed as mean ± SEM unless otherwise indicated. Statistical analyses were performed using GraphPad Prism 10.6.0 (GraphPad Software, La Jolla, CA, USA). Comparisons between two groups were performed using two-tailed Student’s *t*-test; Welch’s correction was applied when variances were unequal. Comparisons among three or more groups were performed using one-way ANOVA followed by Bonferroni’s multiple comparisons test. Data were first assessed for normality. Welch’s ANOVA was used for normally distributed data with unequal variances; the Kruskal–Wallis test was applied for data that did not meet the normality assumption. Alpha diversity indices (Chao1 and Shannon) between groups were compared using the Mann–Whitney U test. Beta diversity was analyzed by principal coordinate analysis (PCoA) based on Bray–Curtis distance, and group differences were assessed by PERMANOVA. Correlations between microbiota and metabolites were assessed using Spearman’s rank correlation coefficient. Statistical significance was granted when the *p* values were <0.05.

## 3. Results

### 3.1. Cold Exposure Elevates Fecal 3-HPA Levels and Exacerbates DSS-Induced Colitis

**Figure 1:**
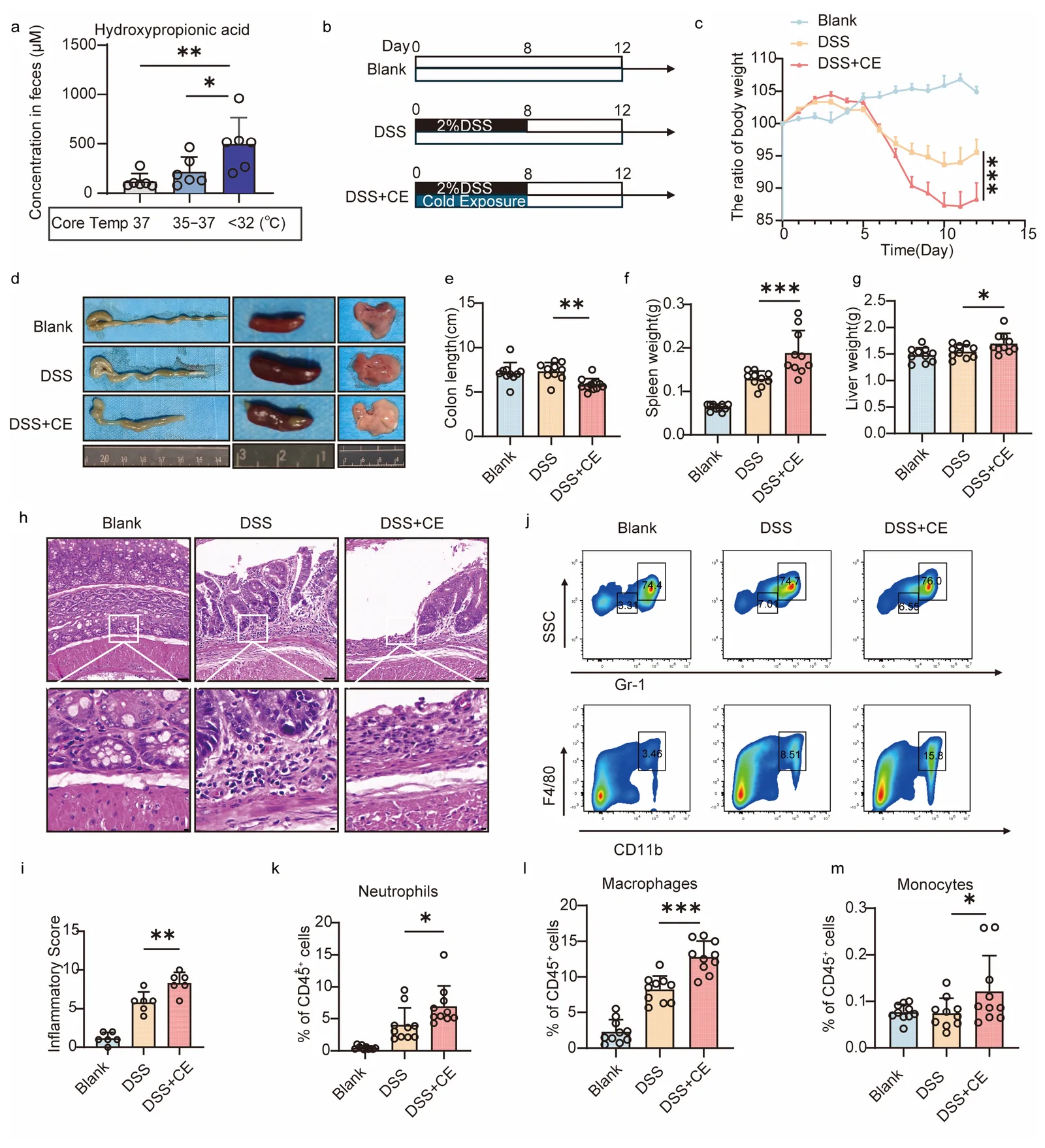
Cold exposure elevates fecal 3-HPA levels and exacerbates DSS-induced colitis associated with altered immune cell infiltration. (**a**) Fecal concentrations of 3-HPA in mice exposed to cold (4 °C) or room temperature (RT), stratified by core body temperature (37 °C, 35–37 °C, and 32 °C groups). (**b**) Experimental design of dextran sulfate sodium (DSS)-induced colitis with or without cold exposure (CE). Mice received 2% DSS and were exposed to cold from day 0 to day 8, followed by monitoring until day 12. (**c**) Body weight changes, presented as percentage of initial body weight, in Blank, DSS, and DSS + CE groups over time. (**d**) Representative gross images of the colon, spleen, and liver from each group. (**e**–**g**) Quantification of colon length (**e**), spleen weight (**f**), and liver weight (**g**) for Blank, DSS, and DSS + CE groups. (**h**) Representative H&E-stained histological images of colon tissues from Blank, DSS, and DSS+CE groups. Lower panels show magnified views of the boxed areas. Scale bars: 100 μm (upper panels) and 20 μm (lower panels). (**i**) Histological inflammatory scores of colon tissues across the groups. (**j**) Representative flow cytometry plots showing the proportions of neutrophils (CD11b^+^ Gr-1hi SSChi), monocytes (CD11b^+^ Gr-1int/low SSClow) and macrophages (CD11b^+^ F4/80^+^) in CD45^+^-gated splenic cells. The gating strategy is provided in Figure S1a. (**k**–**m**) Quantification of the percentages of neutrophils (**k**), macrophages (**l**), and monocytes (**m**) among CD45^+^ cells. Data are presented as mean ± SEM (*n* = 6 per group for (**a**); *n* = 10 per group for (**c**–**m**)). Statistical analyses: one-way ANOVA with Bonferroni’s test, or two-way ANOVA with Tukey’s multiple comparisons test (for body weight curves). * *p* < 0.05; ** *p* < 0.01; *** *p* < 0.001.

To investigate whether cold exposure affects gut microbial metabolites associated with intestinal inflammation, mice were exposed to 4 °C or kept at room temperature, and fecal metabolomes were analyzed. Among the detected metabolites, 3-HPA was markedly increased in cold-exposed mice, with higher levels observed in mice with lower core body temperatures (Figure 1a), indicating an increase correlated with body temperature changes. To determine the impact of cold exposure on colitis severity, mice were subjected to DSS-induced colitis (2% DSS) with or without concurrent cold exposure from day 0 to day 8, followed by monitoring until day 12 (Figure 1b). Mice subjected to DSS-induced colitis with concurrent cold exposure exhibited more pronounced and sustained body weight loss relative to DSS-only controls (Figure 1c). Morphological analysis revealed further shortening of colon length and increased spleen and liver weights (Figure 1d–g), reflecting enhanced systemic inflammation. Histological evaluation showed aggravated epithelial damage, crypt loss, and increased inflammatory cell infiltration in cold-exposed mice, resulting in higher histopathological scores (Figure 1h,i). Flow cytometric analysis demonstrated elevated proportions of neutrophils, monocytes, and macrophages among splenic CD45^+^ cells (Figure 1j–m), consistent with enhanced systemic myeloid mobilization during colitis progression. Consistent with these splenic changes, peripheral blood analysis showed that cold exposure significantly increased circulating monocytes and elevated serum IL-6 and IL-1β levels, whereas circulating neutrophils and serum TNF-α were not significantly changed (Figure S5a–h). Gating strategies for macrophages are provided in Figure S1a. Together, these data indicate that cold exposure increases fecal 3-HPA levels and exacerbates DSS-induced colitis, suggesting a mechanistic link between cold-induced metabolic changes and intestinal inflammation.

### 3.2. 3-HPA Exacerbates DSS-Induced Colitis

**Figure 2:**
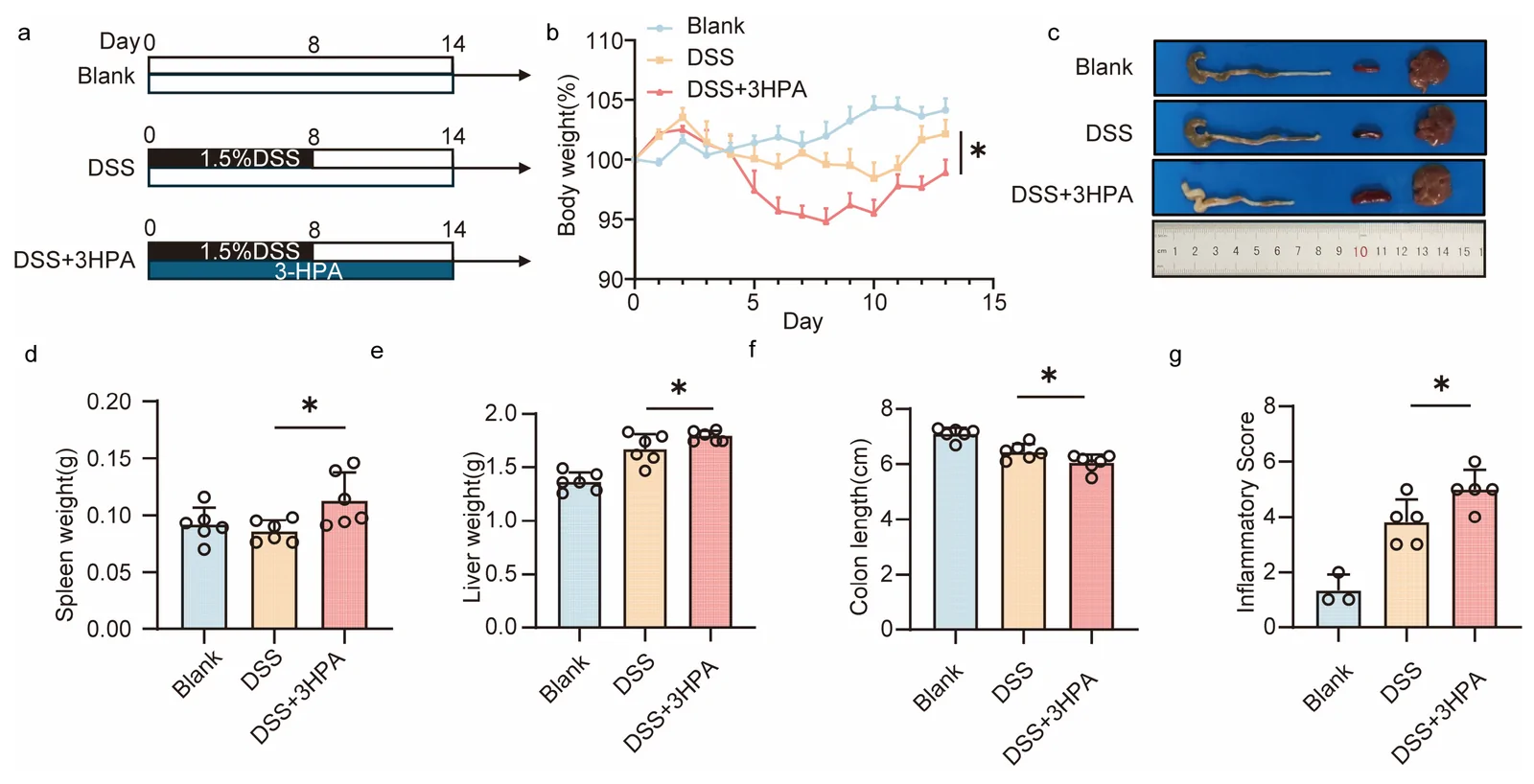
3-HPA supplementation exacerbates DSS-induced colitis. (**a**) Experimental design of DSS-induced colitis with or without 3-HPA supplementation. Mice received 1.5% DSS from day 0 to day 8 and were administered 5 mM 3-HPA in drinking water ad libitum from day 0 to day 14. (**b**) Body weight changes, presented as percentage of initial body weight, in Blank, DSS, and DSS + 3-HPA groups over time. (**c**) Representative gross images of colon, spleen, and liver from each group. (**d**–**f**) Quantification of spleen weight (**d**), liver weight (**e**), and colon length (**f**). 3-HPA treatment increased spleen and liver weights and showed a trend toward reduced colon length compared with DSS alone. (**g**) Histological inflammatory scores of colon tissues. 3-HPA treatment increased histological inflammation compared with DSS alone. Data are presented as mean ± SEM. *n* = 6 mice per group unless otherwise indicated. Statistical analyses: one-way ANOVA with Bonferroni’s test, and two-way ANOVA with Tukey’s multiple comparisons test (body weight curves). * *p* < 0.05.

To determine whether 3-HPA directly contributes to colitis severity, mice were treated with DSS with or without 3-HPA supplementation (Figure 2a). Mice receiving 3-HPA exhibited sustained and more pronounced body weight loss compared with DSS-only controls (Figure 2b). Spleen and liver weights were significantly increased (Figure 2c–e), whereas colon length was modestly reduced (Figure 2f), consistent with enhanced systemic and local inflammation. Histological examination revealed aggravated epithelial damage and inflammatory infiltration in DSS + 3-HPA mice, resulting in significantly higher histopathological scores (Figure 2g). Figure S1c provides additional histological evidence. Together, these results demonstrate that 3-HPA is sufficient to exacerbate DSS-induced colitis.

### 3.3. 3-HPA Supplementation Promotes Immune Infiltration and Impairs Epithelial Barrier Integrity

**Figure 3:**
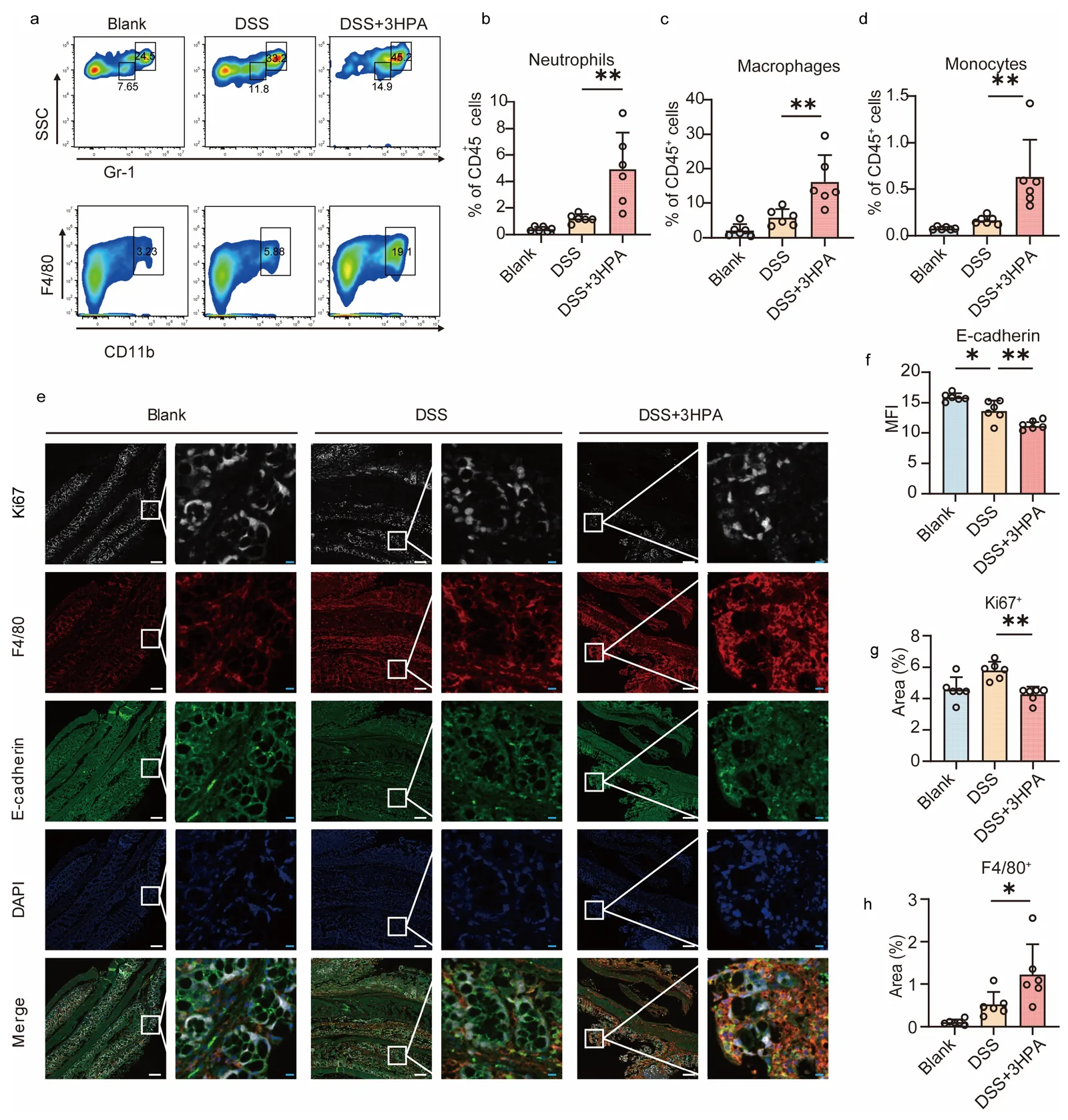
3-HPA supplementation promotes immune infiltration and impairs epithelial barrier integrity. (**a**) Representative flow cytometry plots showing the proportions of neutrophils (CD11b^+^ Gr-1hi SSChi), monocytes (CD11b^+^ Gr-1int/low SSClow) and macrophages (CD11b^+^ F4/80^+^) in CD45^+^-gated splenic cells. The gating strategy is provided in Figure S1b. (**b**–**d**) Quantification of the percentages of neutrophils (**b**), macrophages (**c**), and monocytes (**d**) among CD45^+^ cells. (**e**) Representative immunofluorescence images of colon tissues stained for Ki67, F4/80, E-cadherin, and DAPI. Insets show enlarged views. Scale bars: 100 μm (white) and 10 μm (blue). (**f**–**h**) Quantification of E-cadherin mean fluorescence intensity (MFI) (**f**), Ki67^+^ area (**g**), and F4/80^+^ area (**h**). 3-HPA treatment reduced E-cadherin expression and epithelial proliferation, while increasing macrophage infiltration. Data are presented as mean ± SEM. *n* = 6 mice per group unless otherwise indicated. Statistical analyses: one-way ANOVA with Bonferroni’s test. * *p* < 0.05; ** *p* < 0.01.

Flow cytometric analysis confirmed increased infiltration of neutrophils, monocytes, and macrophages among splenic CD45^+^ cells in DSS + 3-HPA mice compared with DSS controls (Figure 3a–d), supporting enhanced myeloid responses. Representative gating strategies are provided in Figure S1b. In line with the splenic data, 3-HPA treatment significantly increased circulating F4/80^+^ cells and serum IL-6 levels, while circulating neutrophils, monocytes, and serum IL-1β showed increasing trends and serum TNF-α remained unchanged (Figure S5i–p). Immunofluorescence analysis of colonic tissue demonstrated that 3-HPA treatment disrupted epithelial barrier integrity, as evidenced by reduced E-cadherin expression (Figure 3f). In parallel, epithelial proliferative capacity was impaired, as indicated by decreased Ki67^+^ area (Figure 3g), while macrophage infiltration was significantly increased, as shown by elevated F4/80^+^ signal (Figure 3h). Representative staining images are shown in Figure 3e. Collectively, these results indicate that 3-HPA not only enhances systemic myeloid activation in the spleen but also promotes localized macrophage accumulation within the colonic mucosa, thereby contributing to both intestinal inflammation and epithelial barrier impairment.

### 3.4. 3-HPA Disrupts Microbial Metabolic Outputs and Reduces the Production of Short-Chain Fatty Acids

**Figure 4:**
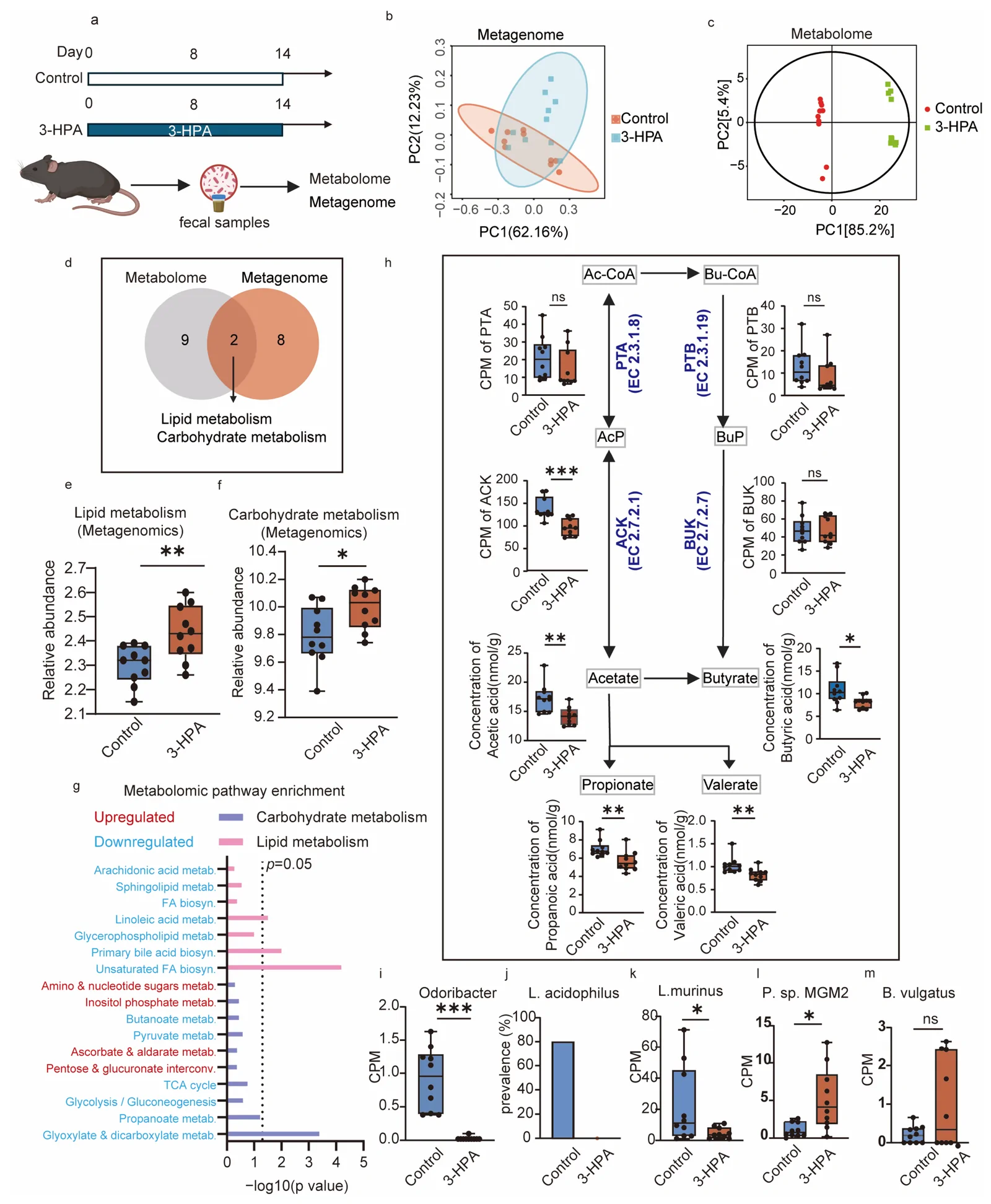
3-HPA reshapes gut microbiome composition and metabolic output, leading to impaired SCFA production. (**a**) Experimental design for paired metagenomic and metabolomic analyses. Mice were given vehicle or 5 mM 3-HPA in drinking water ad libitum for 14 days (*n* = 10 per group). (**b**,**c**) Principal component analysis (PCA) of metagenomic (**b**) and metabolomic (**c**) profiles showing separation between control and 3-HPA-treated mice. (**d**) Venn diagram of overlapping KEGG pathways identified by metagenomic and metabolomic analyses. (**e**,**f**) Relative abundance of lipid metabolism (**e**) and carbohydrate metabolism (**f**) pathways. 3-HPA treatment altered pathways associated with lipid and carbohydrate metabolism. (**g**) Enrichment of KEGG pathways within lipid metabolism and carbohydrate metabolism classes among the overlapping pathways identified by metagenomic and metabolomic analyses. Pathway abbreviations: Arachidonic acid metab., arachidonic acid metabolism; sphingolipid metab., sphingolipid metabolism; FA biosyn., fatty acid biosynthesis; linoleic acid metab., linoleic acid metabolism; glycerophospholipid metab., glycerophospholipid metabolism; primary bile acid biosyn., primary bile acid biosynthesis; unsaturated FA biosyn., unsaturated fatty acid biosynthesis; amino & nucleotide sugars metab., amino sugar and nucleotide sugar metabolism; inositol phosphate metab., inositol phosphate metabolism; butanoate metab., butanoate metabolism; pyruvate metab., pyruvate metabolism; ascorbate & aldarate metab., ascorbate and aldarate metabolism; pentose & glucuronate interconv., pentose and glucuronate interconversions; TCA cycle, tricarboxylic acid cycle; glycolysis/gluconeogenesis, glycolysis and gluconeogenesis; propanoate metab., propanoate metabolism; glyoxylate & dicarboxylate metab., glyoxylate and dicarboxylate metabolism. Pathway regulation was determined based on DA_score values derived from metabolomic analysis. Pathways with positive DA_score are shown in red and indicate upregulation, whereas pathways with negative DA_score are shown in blue and indicate downregulation. (**h**) Quantification of SCFA-related metabolites and associated metabolic intermediates and enzymes, showing reduced SCFA production and decreased acetate kinase following 3-HPA treatment. Abbreviations: Ac-CoA, acetyl-CoA; Bu-CoA, butyryl-CoA; AcP, acetyl phosphate; BuP, butyryl phosphate; PTA, phosphotransacetylase; PTB, phosphate butyryltransferase; ACK, acetate kinase; BK, butyrate kinase. (**i**–**m**) Relative abundance of selected bacterial taxa. 3-HPA treatment decreased beneficial taxa such as *Lactobacillus* spp. and altered other microbial populations. Abbreviations: *L.*, *Lactobacillus*; *P.*, *Prevotella*; *B*., *Bacteroides*. Species names are abbreviated after first mention, including *L. acidophilus* (*Lactobacillus acidophilus*), *L. murinus* (*Lactobacillus murinus*), *P.* sp. *MGM2* (*Prevotella* sp. *MGM2*), and *B. vulgatus* (*Bacteroides vulgatus*). Data are presented as mean ± SEM (*n* = 10 per group). Statistical analysis: unpaired two-tailed Student’s *t*-test with Welch’s correction. ns, not significant; * *p* < 0.05; ** *p* < 0.01; *** *p* < 0.001.

To determine whether 3-HPA directly alters the gut microbial ecosystem, healthy mice were administered 5 mM 3-HPA for 14 days, followed by paired shotgun metagenomic and untargeted metabolomic profiling (Figure 4a). Principal component analysis showed clear separation between control and 3-HPA-treated mice at both microbiome and metabolome levels (Figure 4b,c and Figure S2a–c), indicating a global shift in microbial composition and metabolic output.

Venn diagrams of overlapping KEGG pathways identified by metagenomic and metabolomic analyses revealed that lipid and carbohydrate metabolism pathways were commonly affected (Figure 4d–g), indicating broad reprogramming of microbial metabolic functions. Pathway enrichment analysis based on metabolomic data (Figure 4d) revealed distinct regulation patterns within lipid and carbohydrate metabolism. Specifically, lipid metabolism pathways were predominantly downregulated, including key pathways such as unsaturated fatty acid biosynthesis (unsaturated FA biosyn.), primary bile acid biosynthesis (primary bile acid biosyn.), linoleic acid metabolism (linoleic acid metab.), and glycerophospholipid metabolism (glycerophospholipid metab.), several of which were significantly enriched. Other lipid-related pathways, including arachidonic acid metabolism (arachidonic acid metab.), sphingolipid metabolism (sphingolipid metab.), and fatty acid biosynthesis (FA biosyn.), exhibited weaker enrichment signals.

In contrast, carbohydrate metabolism pathways showed mixed regulation. A subset of pathways, including amino sugar and nucleotide sugar metabolism (amino & nucleotide sugars metab.), inositol phosphate metabolism (inositol phosphate metab.), ascorbate and aldarate metabolism (ascorbate & aldarate metab.), and pentose and glucuronate interconversions (pentose & glucuronate interconv.) exhibited upward trends, but these changes were not statistically significant. Conversely, several central energy-related pathways were downregulated, including glyoxylate and dicarboxylate metabolism (glyoxylate & dicarboxylate metab.), propanoate metabolism (propanoate metab.), the tricarboxylic acid cycle (TCA cycle), pyruvate metabolism (pyruvate metab.), butanoate metabolism (butanoate metab.), and glycolysis and gluconeogenesis (glycolysis/gluconeogenesis), with glyoxylate and dicarboxylate metabolism showing the strongest enrichment signal.

Collectively, these results indicate a broad suppression of lipid metabolic pathways accompanied by selective impairment of core carbon metabolism, suggesting that 3-HPA disrupts microbial energy production and biosynthetic capacity. Consistent with these pathway alterations and the specific downregulation of precursor pathways, 3-HPA treatment significantly reduced the concentrations of major short-chain fatty acids, including acetate, propionate, butyrate, and valerate (Figure 4h).

Metagenomic analysis revealed that key enzymes involved in SCFA biosynthesis were differentially affected, with acetate kinase (ACK) significantly decreased, phosphotransacetylase (PTA) and phosphate butyryltransferase (PTB) showing decreasing trends, and butyrate kinase (BK) remaining relatively stable. These changes indicate that 3-HPA selectively impairs microbial SCFA production, particularly the terminal enzymatic steps for acetate synthesis, reflecting reduced metabolic efficiency. At the taxonomic level, SCFA-producing bacteria such as Lactobacillus acidophilus, Lactobacillus murinus, and *Odoribacter* were decreased, whereas taxa including *Prevotella* sp. *MGM2* and *Bacteroides vulgatus* were enriched (Figure 4i–m). Correlation analyses revealed significant positive associations between SCFA levels and the abundance of these specific taxa, suggesting an association between microbial taxonomic shifts and metabolite output, rather than a direct functional interaction (Figure S2e–g). These findings suggest that the reduction in short-chain fatty acids is linked not only to altered metabolic pathways but also to shifts in the microbial taxa that contribute to their production.

Beyond SCFAs, microbial signaling metabolites were also affected. As shown in Figure S3a, tryptophan metabolism was significantly altered, characterized by decreased levels of multiple indole derivatives, including indole-3-acetic acid, indole-3-propionic acid, indole-3-lactic acid, and indole-3-acrylic acid, while tryptophol levels were increased. Similarly, bile acid metabolism was disrupted, as evidenced by reduced bile salt hydrolase activity and alterations in bile acid-associated microbial taxa, including *Lactobacillus acidophilus* and *Taurinivorans* (Figure S3b–g).

These results demonstrate that 3-HPA administration is associated with broad shifts in the intestinal metabolic environment, characterized by downregulation of lipid-related pathways, impaired central carbon metabolism, and reduced short-chain fatty acid production. Given that SCFAs serve as major energy substrates for intestinal epithelial cells and contribute to epithelial homeostasis, their depletion may create a nutrient-poor intestinal environment. This provides a mechanistic basis for assessing whether 3-HPA directly affects epithelial mitochondrial function and barrier integrity under nutrient-limited conditions.

### 3.5. 3-HPA Impairs Mitochondrial Respiration and Disrupts Junctional Integrity in Intestinal Epithelial Cells

**Figure 5:**
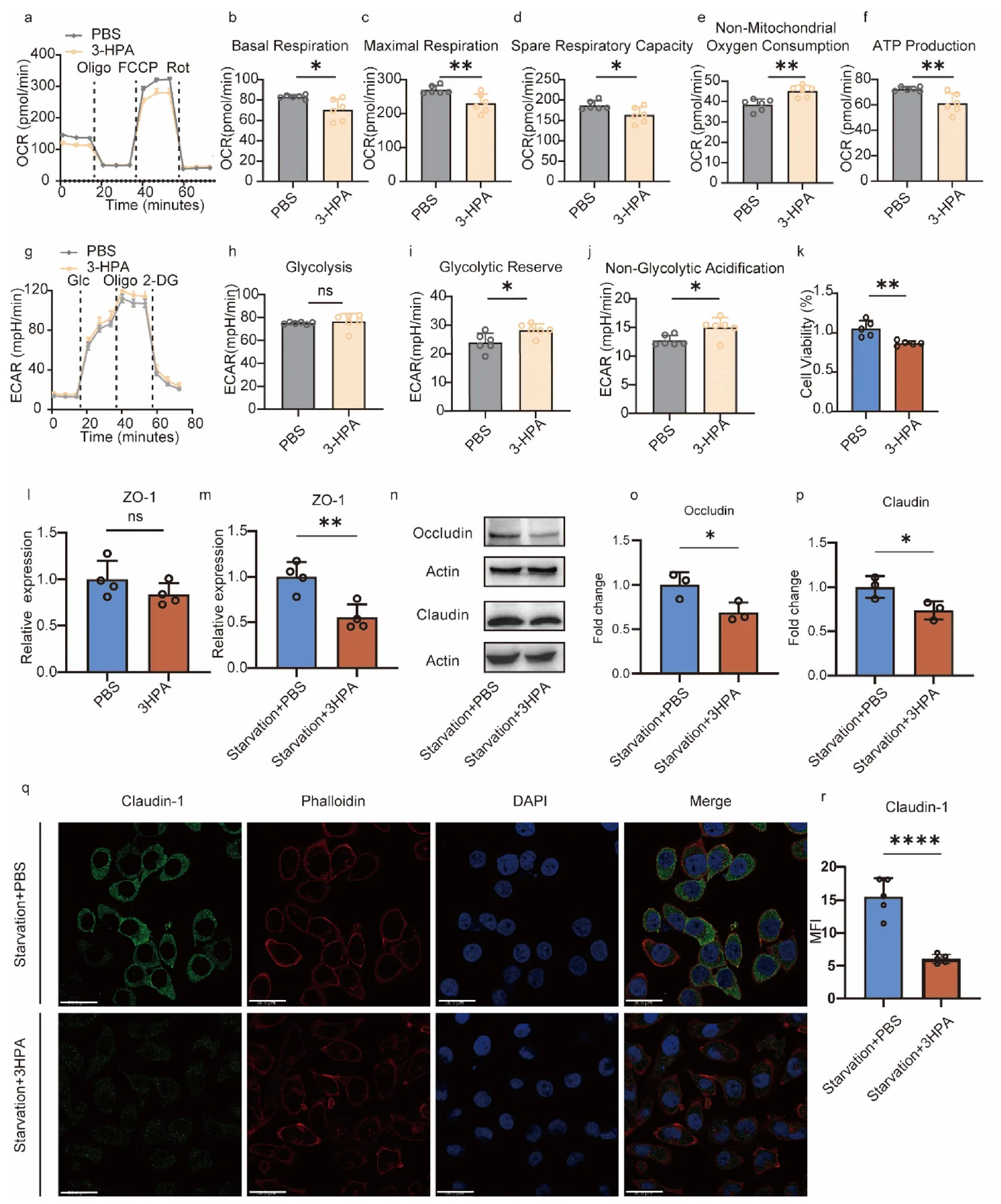
3-HPA impairs mitochondrial respiration and compromises epithelial barrier integrity. (**a**) Representative oxygen consumption rate (OCR) curves in PBS-treated or 5 mM 3-HPA-treated NCM460 cells. (**b**–**f**) Quantification of basal respiration (**b**), maximal respiration (**c**), spare respiratory capacity (**d**), non-mitochondrial oxygen consumption (**e**), and ATP production (**f**). 3-HPA treatment reduced basal respiration, maximal respiration, spare respiratory capacity, and ATP production, while increasing non-mitochondrial oxygen consumption. (**g**) Representative extracellular acidification rate (ECAR) curves in PBS-treated or 5 mM 3-HPA-treated NCM460 cells. (**h**–**j**) Quantification of glycolysis (**h**), glycolytic reserve (**i**), and non-glycolytic acidification (**j**). 3-HPA treatment did not significantly affect glycolysis or glycolytic reserve, but increased non-glycolytic acidification. (**k**) Cell viability following PBS or 5 mM 3-HPA treatment. (**l**,**m**) Relative mRNA expression of *ZO-1* measured by qPCR under basal (**l**) and starvation (**m**) conditions, showing reduced expression following 3-HPA treatment. (**n**) Representative Western blot images of occludin and claudin-1 under starvation conditions. (**o**,**p**) Quantification of occludin (**o**) and claudin-1 (**p**) protein expression. (**q**) Representative confocal images of claudin-1, actin, and DAPI staining under starvation conditions. Scale bar: 40 μm (white). (**r**) Quantification of claudin-1 mean fluorescence intensity (MFI). Data are presented as mean ± SEM from at least three independent experiments. Statistical analysis: unpaired two-tailed Student’s *t*-test. ns, not significant; * *p* < 0.05; ** *p* < 0.01; **** *p* < 0.0001.

Imbalance of gut microbiota and insufficient SCFA production cause epithelial cells to enter a ‘partially starved’ state [16]. Therefore, we further analyzed the effect of 3-HPA on epithelial cells with starvation treatment. First, to determine whether 3-HPA directly affects epithelial bioenergetics, NCM460 cells were treated with 5 mM 3-HPA and analyzed using Seahorse extracellular flux assays. OCR analysis revealed that 3-HPA significantly reduced basal respiration, maximal respiration, spare respiratory capacity, and ATP production, while increasing non-mitochondrial oxygen consumption (Figure 5a–f). ECAR analysis showed that 3-HPA did not affect glycolysis or glycolytic reserve but increased non-glycolytic acidification (Figure 5g–j), indicating selective impairment of mitochondrial oxidative phosphorylation. Consistently, cell viability was reduced following 3-HPA treatment (Figure 5k). Given the SCFA depletion observed in vivo, we next examined the expression of tight junction components in intestinal epithelial cells under starvation conditions. 3-HPA significantly reduced *ZO-1* mRNA expression (Figure 5l,m) and decreased protein levels of the tight junction proteins occludin and claudin-1 (Figure 5n–p). Confocal imaging revealed disrupted tight junction organization and reduced claudin-1 fluorescence intensity (Figure 5q,r). These findings show that 3-HPA impairs mitochondrial oxidative phosphorylation and, under nutrient-limited stress, compromises tight junction barrier integrity in epithelial cells. Although our data do not establish a direct causal sequence between mitochondrial respiration and tight junction regulation, they indicate that 3-HPA affects both bioenergetic and structural pathways in intestinal epithelial cells. Together, these findings support a model in which 3-HPA reshapes the gut microbiota and disrupts microbial metabolic outputs, including SCFA, tryptophan, and bile acid metabolism, thereby creating a metabolically unfavorable intestinal environment. This altered metabolic landscape imposes energetic and signaling stress on epithelial cells, leading to impaired mitochondrial respiration and compromised barrier integrity, ultimately contributing to the exacerbation of intestinal inflammation.

## 4. Discussion

In this study, we identified 3-hydroxypropionate (3-HPA) as a fecal metabolite increased under cold exposure and demonstrated that its exogenous administration is sufficient to aggravate DSS-induced colitis. While cold exposure induces complex, multi-systemic physiological responses, our findings position 3-HPA as a prominent candidate metabolite that contributes to environmentally exacerbated intestinal inflammation. Mechanistically, 3-HPA reshaped gut microbiota composition, disturbed microbial metabolic outputs, and impaired epithelial mitochondrial respiration and barrier integrity. These findings suggest that 3-HPA may serve as a metabolic link between environmental cold stress, gut microbial dysbiosis, and intestinal epithelial dysfunction.

Environmental factors are increasingly recognized as important modulators of intestinal inflammation, but the metabolic intermediates that translate environmental stress into mucosal injury remain incompletely understood [2]. Our data show that cold exposure increased fecal 3-HPA, with higher levels observed in mice with lower core body temperatures. This observation indicates that 3-HPA is responsive to cold-associated physiological changes. More importantly, cold exposure aggravated DSS-induced colitis, as shown by sustained body weight loss, colon shortening, aggravated histological injury, and increased myeloid immune responses. These findings are consistent with the concept that cold stress can worsen intestinal inflammation, but extend this idea by identifying 3-HPA as a candidate metabolite linked to this exacerbation. Although the present study does not prove that 3-HPA is required for cold-induced colitis aggravation, the increase in fecal 3-HPA under cold exposure, together with the effect of 3-HPA supplementation in aggravating colitis, supports a model in which 3-HPA contributes to the inflammatory consequences of cold stress.

A key finding of this study is that 3-HPA alone was sufficient to exacerbate DSS-induced colitis. Compared with DSS treatment alone, 3-HPA supplementation caused more severe body weight loss, increased spleen and liver weights, aggravated histological inflammation, and enhanced infiltration of neutrophils, monocytes, and macrophages. These immune changes suggest that 3-HPA amplifies both local intestinal injury and systemic inflammatory responses. In addition, 3-HPA reduced E-cadherin expression and epithelial proliferation in colonic tissues, while increasing F4/80^+^ macrophage accumulation. Thus, the pathological effect of 3-HPA is not limited to immune activation; it is also associated with impaired epithelial repair and disruption of epithelial integrity. This point is important because epithelial barrier dysfunction is not merely a consequence of colitis, but also a driver of persistent mucosal inflammation [17]. Our multi-omics analyses further revealed that 3-HPA administration is associated with broad changes in the gut microbial ecosystem. At both metagenomic and metabolomic levels, 3-HPA-treated mice were clearly separated from controls, indicating that 3-HPA reshapes microbial composition and metabolic output even in the absence of DSS-induced inflammation. Among the altered pathways, tryptophan metabolism was notably affected. Although the tryptophan metabolism pathway was enriched in the 3-HPA group, tryptophanase abundance was decreased, and several indole derivatives, including indole-3-acetic acid, indole-3-propionic acid, indole-3-lactic acid, and indole-3-acrylic acid, were reduced, whereas tryptophol was increased. This pattern suggests that global enrichment of a pathway does not necessarily indicate the production of protective downstream metabolites. Instead, 3-HPA administration is accompanied by a shift in tryptophan metabolite profiles away from indole production toward alternative products, which correlates with the observed taxonomic remodeling. Because indole derivatives contribute to epithelial homeostasis and mucosal immune regulation, their reduction may weaken microbial support for barrier maintenance [18,19]. Bile acid metabolism was also altered by 3-HPA treatment. The bile acid profile changed, and the abundance of bile salt hydrolase was reduced, particularly that attributed to *Lactobacillus acidophilus*. Since bile salt hydrolase activity participates in bile acid deconjugation and influences the generation of secondary bile acids, these findings suggest a potential link between 3-HPA exposure and altered microbial bile acid transformation [20,21]. Furthermore, while the altered abundance of taxa such as Taurinivorans and L. acidophilus coincides with these metabolic shifts, we emphasize that these multi-omics correlations do not directly prove that these specific taxa are solely responsible for the observed bile acid or tryptophan alterations without targeted functional validation. Together with the tryptophan metabolic changes, these data indicate that 3-HPA does not simply shift microbial taxonomy; it changes the metabolic functions through which the microbiota communicates with the host epithelium.

The reduction in short-chain fatty acids provides another important mechanism linking microbial dysregulation to epithelial injury. Integrated pathway analysis showed that lipid metabolism and carbohydrate metabolism were broadly affected by 3-HPA. In particular, lipid metabolic pathways were mainly downregulated, while several central carbon metabolism pathways, including propionate metabolism and the tricarboxylic acid cycle, were reduced. Consistent with these pathway changes, 3-HPA decreased the levels of acetate, propionate, butyrate, and valerate. The decrease in acetate kinase, together with decreasing trends in phosphotransacetylase and phosphate butyryltransferase, suggests impaired short-chain fatty acid biosynthesis, especially in acetate-related enzymatic processes. At the taxonomic level, bacteria associated with short-chain fatty acid production, including *Lactobacillus acidophilus*, *Lactobacillus murinus*, and *Odoribacter*, were reduced [22]. Correlation analyses further supported the association between these microbial changes and short-chain fatty acid depletion. These findings suggest that 3-HPA reduces both the microbial capacity and the metabolic output required to maintain a short-chain fatty acid-rich intestinal environment.

The consequences of these metabolic alterations for epithelial cells were supported by our in vitro experiments. Intestinal epithelial cells rely heavily on mitochondrial oxidative phosphorylation to sustain energy-demanding processes such as barrier maintenance, junctional remodeling, and epithelial repair [23]. In NCM460 cells, 3-HPA markedly reduced basal respiration, maximal respiration, spare respiratory capacity, and ATP production, while increasing non-mitochondrial oxygen consumption. In contrast, glycolysis and glycolytic reserve were not increased, indicating that epithelial cells did not mount an effective glycolytic compensation. This pattern suggests that 3-HPA directly impairs epithelial mitochondrial function. The reduction in cell viability further supports the biological relevance of this bioenergetic defect. The barrier assays further connect microbial metabolic disruption to epithelial dysfunction. Because 3-HPA reduced short-chain fatty acid availability in vivo, we assessed epithelial barrier markers under nutrient-limited conditions. Under this condition, 3-HPA reduced *ZO-1* expression and decreased the protein levels of occludin and claudin-1. Confocal imaging also showed reduced claudin-1 fluorescence intensity and disrupted junctional organization. These findings suggest that 3-HPA makes epithelial cells more vulnerable when nutrient support is limited. In the context of the in vivo data, the combined loss of short-chain fatty acids, reduction in indole derivatives, and changes in bile acid metabolism may create an intestinal environment in which epithelial cells receive insufficient metabolic and signaling support. Under such conditions, direct mitochondrial impairment by 3-HPA may further compromise barrier integrity and promote inflammation.

Our findings extend current understanding of microbiota-derived metabolites in intestinal inflammation. Many studies have focused on protective metabolites, such as short-chain fatty acids and indole derivatives, whereas fewer have examined microbial metabolites that may actively promote mucosal injury [24]. Previous work has shown that 3-HPA can participate in host–microbe interactions and influence immune responses [12]. Our study expands this concept by showing that 3-HPA can also reshape microbial metabolism and directly impair epithelial function. Thus, 3-HPA may act at multiple levels: altering microbial community structure, reducing beneficial metabolic outputs, disrupting epithelial bioenergetics, and weakening barrier integrity. This multilayered effect may explain why 3-HPA supplementation aggravated DSS-induced colitis. Several limitations should be noted, which may affect the universality of our conclusions. Notably, our in vivo experiments exclusively utilized male mice; because sex differences can significantly influence immune responses and gut microbiota composition, the current findings may not be universally applicable to females, and future studies incorporating dual-sex cohorts are needed to overcome this biological bias. First, although cold exposure increased fecal 3-HPA and 3-HPA supplementation worsened colitis, the present study establishes association rather than absolute causation. Given that cold stress triggers widespread physiological, endocrine, immune, and metabolic alterations, we cannot completely rule out the contribution of other cold-induced systemic responses. Future studies utilizing targeted interventions to specifically block or deplete 3-HPA production during cold exposure will be essential to prove its obligatory role. Second, the metagenomic and metabolomic analyses rely on pathway enrichment and correlation analyses. While these multi-omics data reveal strong statistical associations among microbial taxa, enzymes, and metabolite shifts, they inherently cannot distinguish mathematical correlation from direct functional interaction or causal regulation. Consequently, gnotobiotic models, targeted bacterial supplementation, or targeted microbial enzyme manipulation are required in future studies to dissect these relationships. Third, because 3-HPA was administered via drinking water, the observed gut microbial alterations may reflect a combination of direct microbial responses to 3-HPA and indirect consequences of altered host physiology, nutrient availability, or disease progression. Future in vitro co-culture of defined bacterial strains with 3-HPA, together with gnotobiotic mouse models, will be needed to separate direct microbial effects from secondary host-mediated effects. Fourth, although 3-HPA impaired both mitochondrial oxidative phosphorylation and tight junction expression/organization in intestinal epithelial cells, these parameters were measured in separate assays. We did not perform interventional experiments, such as mitochondrial rescue or respiration inhibition, to establish the two observations within the same mechanistic sequence. Whether barrier dysfunction is a downstream consequence of mitochondrial impairment or a parallel toxic effect of 3-HPA therefore remains to be determined. Fifth, although our study evaluated systemic myeloid responses via splenic flow cytometry and local colonic inflammation via in situ F4/80 immunofluorescence, we did not perform flow cytometric profiling of colonic lamina propria immune cell populations. Future studies characterizing these gut-resident immune subsets will help clarify the immunological crosstalk between systemic myeloid mobilization and intestinal pathology. Finally, the translatability of these murine findings to human pathology remains to be determined. In particular, it will be important to test whether 3-HPA is elevated in patients with IBD or in individuals exposed to cold stress.

## 5. Conclusions

In conclusion, this study identifies 3-HPA as a cold-associated microbial metabolite that exacerbates DSS-induced colitis. 3-HPA disrupts gut microbial metabolic outputs, including short-chain fatty acids, tryptophan-derived indole metabolites, and bile acid-related pathways, while directly impairing epithelial mitochondrial respiration and barrier integrity. These findings support a model in which environmental cold stress promotes intestinal inflammation through microbial metabolic reprogramming and epithelial energy stress. Targeting 3-HPA production or its downstream epithelial effects may provide a potential strategy for limiting environmentally exacerbated intestinal inflammation.

## Data Availability

The raw data presented in this study can be found in the NCBI repository with accession number PRJNA1480396.

## Supplementary Materials

The following supporting information can be downloaded at https://www.mdpi.com/article/10.3390/metabo16080592/s1, Figure S1: Gating strategies for splenic immune-cell subsets and representative colonic histology. Figure S2: Extended microbiome and metabolome analyses following 3-HPA treatment. Figure S3: 3-HPA alters tryptophan metabolism and bile acid profiles in healthy mice. Figure S4: 3-HPA alters fatty acid oxidation-associated mitochondrial respiration and gene expression. Figure S5: Circulating myeloid cell populations and serum cytokine levels in the two colitis cohorts.

## Abbreviations

The following abbreviations are used in this manuscript:

IBD	Inflammatory bowel disease
3-HPA	3-hydroxypropionate
DSS	dextran sulfate sodium
UC	ulcerative colitis
CD	Crohn’s disease
SCFAs	short-chain fatty acids
AhR	aryl hydrocarbon receptor
OCR	Oxygen consumption rate
ECAR	Extracellular acidification rate
ACK	acetate kinase
PTA	phosphotransacetylase
PTB	phosphate butyryltransferase
BK	butyrate kinase
IAA	indole-3-acetic acid
IPA	indole-3-propionic acid
ILA	indole-3-lactic acid
IAcrA	indole-3-acrylic acid
IEA	tryptophol
DCA	Deoxycholic acid
THCA	Taurohyocholic acid
TDCA	Taurodeoxycholic acid
TLCA	Taurolithocholic acid
MDCA	Murideoxycholic acid
FCCP	Carbonyl cyanide-4-(trifluoromethoxy)phenylhydrazone
